# The Functionality of Mobile Apps for Anxiety: Systematic Search and Analysis of Engagement and Tailoring Features

**DOI:** 10.2196/26712

**Published:** 2021-10-06

**Authors:** Andreas Balaskas, Stephen M Schueller, Anna L Cox, Gavin Doherty

**Affiliations:** 1 School of Computer Science and Statistics Trinity College Dublin Dublin Ireland; 2 Department of Psychological Science University of California, Irvine Irvine, CA United States; 3 Department of Informatics University of California, Irvine Irvine, CA United States; 4 UCL Interaction Centre University College London London United Kingdom

**Keywords:** mental health, cognitive behavioral therapy, mobile apps, anxiety, stress, mHealth, mobile phone

## Abstract

**Background:**

A range of mobile apps for anxiety have been developed in response to the high prevalence of anxiety disorders. Although the number of publicly available apps for anxiety is increasing, attrition rates among mobile apps are high. These apps must be engaging and relevant to end users to be effective; thus, engagement features and the ability to tailor delivery to the needs of individual users are key. However, our understanding of the functionality of these apps concerning engagement and tailoring features is limited.

**Objective:**

The aim of this study is to review how cognitive behavioral elements are delivered by anxiety apps and their functionalities to support user engagement and tailoring based on user needs.

**Methods:**

A systematic search for anxiety apps described as being based on cognitive behavioral therapy (CBT) was conducted on Android and iPhone marketplaces. Apps were included if they mentioned the use of CBT for anxiety-related disorders. We identified 597 apps, of which 36 met the inclusion criteria and were reviewed through direct use.

**Results:**

Cognitive behavioral apps for anxiety incorporate a variety of functionalities, offer several engagement features, and integrate low-intensity CBT exercises. However, the provision of features to support engagement is highly uneven, and support is provided only for low-intensity CBT treatment. Cognitive behavioral elements combine various modalities to deliver intervention content and support the interactive delivery of these elements. Options for personalization are limited and restricted to goal selection upon beginning use or based on self-monitoring entries. Apps do not appear to provide individualized content to users based on their input.

**Conclusions:**

Engagement and tailoring features can be significantly expanded in existing apps, which make limited use of social features and clinical support and do not use sophisticated features such as personalization based on sensor data. To guide the evolution of these interventions, further research is needed to explore the effectiveness of different types of engagement features and approaches to tailoring therapeutic content.

## Introduction

### Background

Anxiety disorders are the most common type of mental health problem. Several studies have shown that cognitive behavioral therapy (CBT) is an effective treatment for anxiety disorders [1-4]. CBT focuses on a person’s cognitive processes (thoughts, images, beliefs, and attitudes) and behavior to recognize and address negative thinking patterns and beliefs. Depending on the specific anxiety disorder, different CBT techniques are weighted differently during therapy [3]. Unfortunately, only a minority of individuals with an anxiety disorder have access to treatment [5-7].

Internet adoption and advances in technology have led to an increase in research on internet-delivered CBT to make evidence-based psychotherapy more accessible and cost-effective [8,9]. Internet-delivered CBT is delivered via desktop computers, laptops, or tablets to help patients build core CBT knowledge and skills while reducing reliance on traditional face-to-face sessions [8]. Mobile apps provide a promising avenue for increasing access to mental health interventions. Apps can be used to deliver a range of intervention strategies, provide information about mental health, and enable real-time communication with health care professionals. Several studies have examined the effectiveness of delivering interventions using such apps [10,11]. These apps are likely to become increasingly sophisticated, and advances in technology have opened up possibilities for the delivery of *just-in-time adaptive interventions* that aim to provide the most beneficial interventions based on data collected from sensors or provided by users [12]. This creates opportunities for the delivery of deeply personalized interventions [13].

Although the number of mental health apps available is increasing, there are high attrition rates among mobile apps [14-16]. High attrition rates may be attributed to a lack of knowledge regarding the translation of treatment elements in CBT into engaging digital elements, the loss of a therapist-client relationship, the lack of individualized treatment, and the omission of important therapeutic components [8,17,18]. Hence, we need to understand the content in mental health apps from the viewpoint of not only evidence-based strategies but also of engagement elements and other features relevant to digital delivery.

### Reviewing Mental Health Apps

The increased availability of mental health apps has motivated researchers to create guidelines for assessing such apps [19-22]. In addition, several papers have recently reviewed apps targeting a variety of mental health conditions, including depression [23-26], bipolar disorder [27], and anxiety disorders [28-33]. Previous reviews targeting anxiety disorders have aimed to assess the extent to which mobile apps are grounded in theory [30,31] or evaluate the extent of expert involvement in development [28,29,32]. Several reviews have also categorized different types of mobile apps [25,28] and identified the range and frequency of treatment elements available [34]. The results from these reviews have indicated that mobile apps for anxiety do not provide information on the source of their content [28] and often lack the involvement of health care professionals in their development [29]. Most apps are inconsistent with evidence-based treatment [30-32] and lack published studies on effectiveness [28]. The examination of sensors used to collect data or provide support based on collected data has received little attention among these studies. Only 1 app review that we are aware of has investigated the use of sensors—in a review of apps targeting children and adolescents with anxiety disorders [31].

A review that examined common treatment elements within depression and anxiety apps found that core treatment elements (eg, exposure and restructuring) were rarely included [34]. Looking beyond anxiety, CBT apps for depression offer an eclectic mix of features, including many that are not evidence-based. The apps offer limited CBT features—with their presence or absence not linked with expert involvement in app creation—and lack elements used in high-intensity interventions [23]. A study found that the utility of self-help CBT or behavioral activation apps is questionable, and their usability is highly variable; furthermore, apps are rarely accompanied by privacy policies [26]. Most apps targeting depression provide multiple functions [24,25], provide an interactive interface, and use text as the main type of media [25]. Similarly, the content of apps targeting bipolar disorder is not in line with practice guidelines, and most apps lack citations and privacy policies [27]. Consequently, the case for recommendation of available apps to users is not as strong as it could be.

The design of usable and effective mental health apps is a key challenge and depends on an understanding of what works, for whom, and under which circumstances. This requires a greater understanding of what these apps actually provide and what strategies they employ to engage and deliver treatment to users. Thus, there is a need to examine both the provision of features that encourage regular use and tailoring of content delivery to provide interaction that is both engaging and effective for particular users. As the concept of engagement research is broad and depends on the context that will be examined [35], we define engagement features as functionality that encourages regular use, makes app content more appealing, and in general, helps users to stay engaged with therapy or the app itself. Tailoring in the context of technology-based interventions refers to the adjustment of technology-delivered self-help programs to suit the user’s needs, characteristics, and comorbidities of case formulation [36,37]. Within the context of anxiety apps, this relates primarily to the tailoring of content to specific user groups and needs.

Within the academic literature, the use of functionality based on sensors is seen as a key strategy for delivering more effective content in future systems [12,38-41]; therefore, any use of such features is of interest.

### Objectives

The objectives of this study are to explore the functionality of publicly available mobile apps for anxiety that integrate CBT, with a focus on content delivery, including engagement and tailoring features. Although various studies have explored the evidence base of and evidence-based content in anxiety apps [8,34,42], to our knowledge, no review of apps has been conducted exploring content delivery in apps for anxiety disorders.

## Methods

### Overview

Apps were identified through a systematic search of the two most widely used platforms: Android’s Google Play Store and Apple’s iOS App Store [16]. Initial searches were conducted in February 2020 to explore the inclusion criteria and analysis methodology. The corpus presented here is based on a final search conducted in June 2020. The search was based on the following keywords related to anxiety disorders: *anxiety*, *stress*, *worry*, *phobia*, and *panic*. In addition, we searched for the following keyword related to CBT: *cognitive behavioural*. Separate searches for each of these keywords were conducted on each app store and applied across all categories of apps.

Studies suggest that only a small number of users look beyond the first 10 ranked apps [43]. In addition, the first five apps for a given search term are the most downloaded [43]. Therefore, as a conservative approximation of those visible to potential users, only the first 50 results were included from each unique search. Search results for each of these keywords were automatically downloaded using scripts from the US version of Google Play and Apple’s App Store [44,45]. Recorded information included app name, description, price, developer name, average rating, and the number of user ratings.

### Selection Criteria

**Figure 1:**
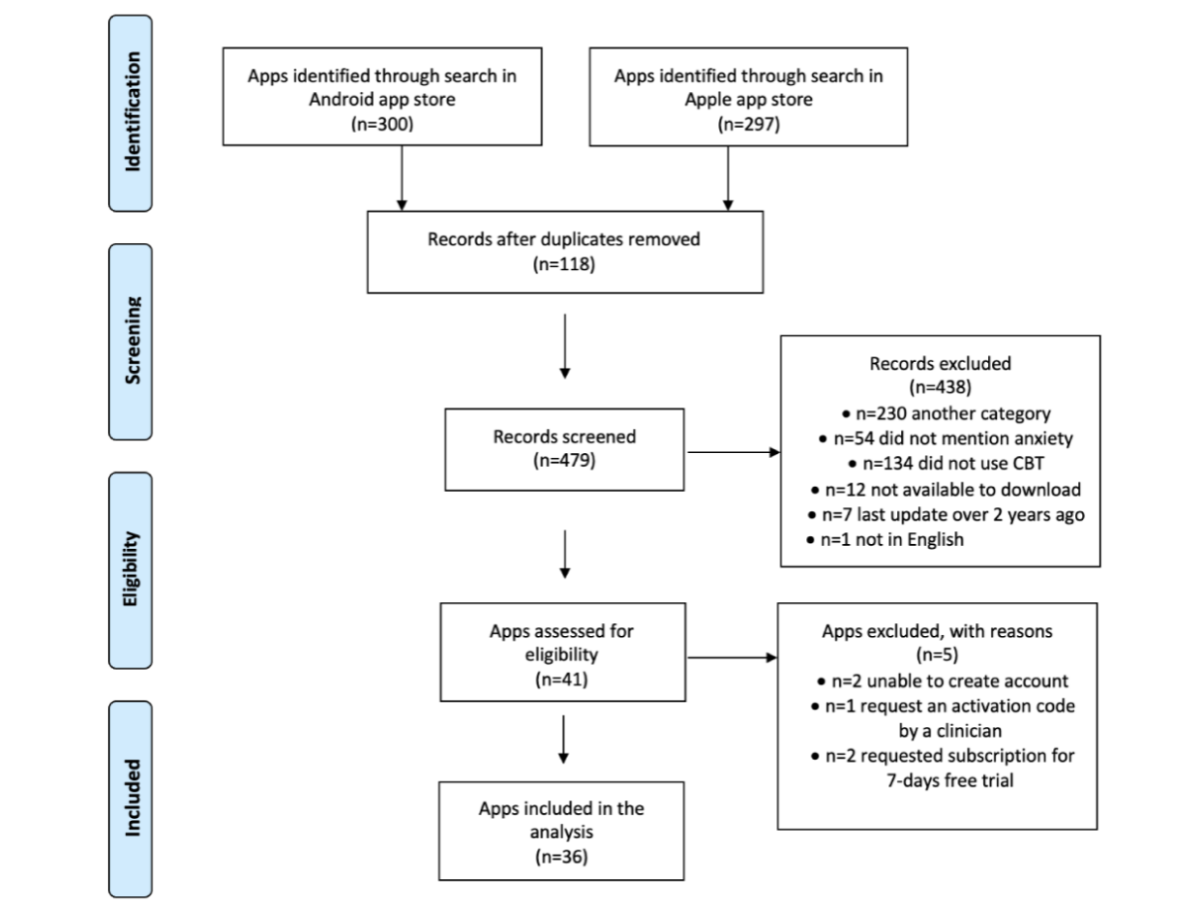
App selection process. CBT: cognitive behavioral therapy.

We included apps that met the following criteria: (1) the app was in the health and fitness or medical category of the app store, (2) mentioned the use of CBT in the title or store description when we searched for anxiety-related keywords, (3) mentioned anxiety-related disorders when we searched for “cognitive behavioural,” (4) were currently available to download, and (5) were available in English. Apps were excluded if (6) they did not mention the treatment or management of anxiety-related symptoms in their description pages and (7) were last updated more than two years ago. We downloaded apps that were free or offered in-app purchases to have the broadest appeal because studies suggest that free apps are more likely to be downloaded than paid apps [46]. We coded both the free and premium features of these apps. Two reviewers reviewed a pilot sample of apps to clarify the inclusion criteria before proceeding. One reviewer independently applied the inclusion criteria. The study selection process is illustrated in Figure 1.

### Data Extraction and Coding

Apps meeting the inclusion criteria were downloaded onto either a Samsung Galaxy S9 Plus (Android version 9) or an iPhone 8 Plus (iOS version 14.0). The American Psychiatric Association model for assessing mobile mental health apps was used as a starting point [19]. Descriptive characteristics related to the following features were extracted from the marketplaces: general background information (store, price, target audience, popularity, privacy and safety, accessibility, claimed scientific underpinning, and medical disclaimer). We assessed accessibility by manually checking each app to identify options relevant to visual, auditory, or motor impairment or explicitly termed accessibility options within the apps.

Engagement features were extracted using a classification from a review by Stawarz et al [23] on the functionality of CBT apps for depression. They examined the functionality of CBT apps for depression by recording both therapeutic and engagement features from their description pages. We recorded features that encourage regular use, make app content more appealing, and in general, help users to stay engaged with therapy or the app itself. In addition, we recorded CBT therapeutic features; 2 researchers independently indicated whether a given feature type represented a CBT component used in the treatment of anxiety, similar to other efforts used to characterize evidence-based app content [26,34,47]. In cases of disagreement, discussions between the authors were conducted until full agreement was established before proceeding. See Textbox 1 for definitions of how components were operationalized for study coders.

**Evidence-based treatment components within study apps.**

**Psychoeducation**

- Education about anxiety (definition, description of cycle of reinforcement, description of symptoms)
- Education on the cognitive behavioral model

**Self-monitoring**

- Monitoring cognition
- Monitoring emotions or symptoms
- Monitoring behaviors

**Cognitive techniques**

- Identifying thoughts
- Cognitive restructuring

**Behavioral techniques**

- Behavioral activation
- Behavioral experimentation

**Relaxation skills**

- Mindfulness exercises
- Progressive muscle relaxation
- Breathing exercises

A sample of apps was reviewed to identify functionality features; 10 apps were then open-coded to create the initial codebook. The codebook was refined following discussion with all authors, and all the apps were coded to the refined scheme. In cases of disagreement, discussions between the authors were conducted until full agreement was established before proceeding.

We coded general characteristics for each app, including target audience, popularity, privacy and safety, accessibility, claimed scientific underpinnings, and medical disclaimers. We coded six main types of functionality and recorded the engagement features—screening, self-monitoring, visualization of data entries, gamification, social features and support (immediate support and crisis support), and features used to deliver CBT treatment elements. We also recorded CBT treatment components, including psychoeducation, cognitive techniques, behavioral techniques, and relaxation skills.

In addition, we recorded tailoring features that aim to personalize treatment, for example, by delivering content targeted to individual user needs or allowing users to customize content based on their needs. We distinguished personalization and customization based on the nature of user involvement. Personalization is used to tailor the user’s experience based on their previous behaviors, whereas customization is initiated by users and allows the modification of app features based on their preferences. The codes covered five main types of tailoring functionality: interface customization, treatment-oriented customization, content tailoring offered immediately after installation (app-driven tailoring), tailoring based on self-monitoring entries (mood-driven personalization), and customization of push notifications. We also recorded (1) the use of sensors and (2) the provision of support based on sensor data. Textbox 2 lists the features identified in the apps in a hierarchical manner. See Textbox 3 for the classification of functionality and engagement features.

**Hierarchical organization of the results section.**

**General characteristics**

- Target audience
- Popularity
- Privacy and safety
- Accessibility
- Claimed scientific underpinning
- Medical disclaimer

**Functionality**

- Screening
- Self-monitoring
- Visualization of data entries
- Gamification
- Social features and support
  Immediate support
  Crisis support
- Features used for delivery of cognitive behavioral therapy treatment elements
  Psychoeducation
  Cognitive techniques
  Behavioral techniques
  Relaxation skills

**Tailoring features**

- Interface customization
- Treatment-oriented customization
- App-driven tailoring
- Mood-driven personalization
- Customization of push notifications

**Functionality types and engagement features.**

**Functionality and engagement features**

- Screening
- Self-monitoring
- Data visualization: graphs and charts, reports supporting graphs and charts
- Gamification: games and gamification
- Tailoring: customization options, notifications, and reminders
- Social features and support: ability to share data with others, peer support, and ability to contact a therapist
- Features used for delivery of cognitive behavioral therapy treatment elements: chat with a bot, treatment program format, ability to add pictures and videos, audio content, video content, and question and answer interface

## Results

### General Characteristics

Of the 479 unique apps screened, 36 met the inclusion criteria (Figure 1), in a range similar to that found in other reviews [27,32]; 11 were available on the Android platform, 4 on the Apple Store, and 21 on both platforms. One app offered an Apple smartwatch version. The apps spanned two categories: health and fitness (26/36, 72%) and medical (10/36, 28%). None of the apps focused on a specific anxiety disorder. In total, 14 apps were free to download (14/36, 39%), and the rest (22/36, 61%) were free with in-app purchases. In-app purchases were offered mostly as monthly or yearly subscriptions (21/22, 95%). One of these apps offered the option of lifetime subscription. Most apps identified through the app stores were last updated in the previous 6 months (27/36, 75%), and only nine apps were last updated before that period.

### Target Audience

Both marketplaces provide formal age classification. The majority of Android apps in the sample were classified as being suitable for children ≥3 years (31/32, 97%), and only 1 app recommended parental guidance. Most apps (15/25, 60%) in the Apple Store were classified as being suitable for adolescents ≥12 years, 32% (8/25) for children ≥4 years, and 8% (2/25) for adolescents ≥17 years. In addition, 1 app recommended use for ages between 11 and 19 years and allowed use by younger children with the support of a carer, although it was classified as being suitable for children ≥3 years (Android) and 4 years (Apple).

### Popularity

Most apps provided a rating score in the marketplace (33/36, 92%). The rating for most apps (out of 5 stars) was above 4.0 (26/33, 79%). Multimedia Appendix 1 provides information on app ratings. Two apps in the Android marketplace received an editor’s choice award and another received a standout well-being app award.

### Privacy and Safety

Privacy policies were available, either in the app or as a link from the app store description for most apps (34/36, 94%). Two apps lacked a privacy policy, which means no protection for personal information or safeguards against misuse of mental health data. The privacy policy for 1 app was not in English. Of all the apps with a privacy policy, an account or password creation was mandatory for 9 (out of 35, 26%) and optional for 1 (out of 35, 3%). A total of 15 apps provided the option to set up a personal identification number (13/15, 87%) or biometric authentication (2/15, 13%). The setup for password protection was offered in the premium version for one app. The remaining apps did not provide any security features to restrict access to the data.

### Accessibility

Most of the apps required an internet connection to function (24/36, 67%), and a small number (3/36, 8%) provided reduced functionality without an internet connection, which may disadvantage those without a reliable connection. Accessibility options for those with impaired vision or other disabilities were offered by only 2 apps (out of 36, 5%). One of these apps allowed the text size to be changed. The other app (Happify) offered a variety of options, including compatibility with assistive technology (such as voice assistants), high-contrast mode for those with low vision or color blindness, accessibility warnings for activities that require visual or audio interactions, font resize support for low-vision users, and the option to disable animations.

### Claimed Scientific Underpinning

All the apps claimed in their description page to be designed based on validated psychological treatments. A total of 20 apps were designed to provide techniques based on CBT (20/36, 56%). The remainder integrated CBT techniques combined with other psychological treatment approaches, including positive psychology (5/36, 14%), acceptance and commitment therapy (4/36, 11%), and dialectical behavior therapy (3/36, 8%).

### Medical Disclaimer

Most apps provided a medical disclaimer, indicating that the app was not a replacement for clinical treatment (21/36, 58%). A total of 15 apps did not provide any disclaimer on the marketplace or app’s website. In addition, 10 apps made the disclaimer easy to find and read by presenting it on the description page of the app on the marketplace (8/10, 80%) or when downloading the app (2/10, 20%). The remainder presented the disclaimer in their terms of use (4/11, 36%), on the app menu (5/11, 45%), or in the *frequently asked questions* section (2/11, 18%).

### Functionality Analysis

#### Functionality Types

The following section discusses the functionality of the identified apps, with a focus on engagement and tailoring features. For all apps, we recorded details of features to support user engagement with therapy or the app itself offered in the free (Table 1) and premium versions (Table 2).

**Table 1:** Engagement features.

App name	Visualization	Gamification	Customization	Social	Chatbot
Bloom	✓^a^		✓		
CBT Companion	✓		✓	✓	
CBT diary	✓		✓	✓	
CBT Journey	✓			✓	
CBT MH	✓				
CBT diary	✓			✓	
CBT Tools	✓	✓	✓		
ClearFear	✓	✓	✓		
CBT	✓				
CBT (2)	✓				
De-stressMe	✓	✓			
ezeCBT	✓		✓	✓	
FearTools	✓				
FreeCBT	✓		✓		
Happify	✓	✓	✓	✓	✓
Innerhour	✓	✓	✓		✓
Life	✓		✓		✓
Mindease	✓		✓		
Mindshift	✓		✓	✓	
Moodfit	✓		✓	✓	
Moodnotes	✓		✓	✓	
Moodpath	✓		✓	✓	✓
MoodSpace	✓		✓	✓	
Panic Pit Stop					
Pocketcoach	✓				✓
Reflectly	✓		✓	✓	
Sanvello	✓	✓	✓	✓	✓
Stress & Anxiety Companion	✓		✓		
Thoughts	✓				
UpLift	✓	✓	✓		
What’s up	✓		✓	✓	
Woebot	✓		✓		✓
WorryKit	✓				
WorryTree	✓		✓	✓	
Wysa	✓		✓		
Youper	✓		✓	✓	

^a^Feature present.

**Table 2:** Engagement features available in the premium version of apps.

App name	Therapist	Program	Data visualization	Reports	Data sharing	Support	Other
Bloom		✓^a^	✓	✓			
CBT diary			✓		✓	✓	✓
CBT Companion		✓		✓	✓	✓	✓
Happify		✓	✓	✓			
Innerhour	✓	✓		✓		✓	
Mindshift	✓	✓	✓	✓			
Moodnotes			✓				
Moodpath		✓	✓			✓	
Pocketcoach	✓	✓	✓			✓	
Reflectly			✓				
Sanvello		✓	✓				✓
UpLift		✓					
What’s up							✓
WorryTree				✓			✓
Wysa	✓	✓				✓	
Youper							✓

^a^Feature present.

#### Screening

In total, 12 (12/36, 33%) apps offered functionality to screen for a variety of psychological disorders using questionnaires. The screening was user-initiated for five apps. The remaining apps provided screening when downloading the app (5/12, 42%) or during app use (2/12, 17%). The purpose of the screening was to help users track and manage their progress and provide insights. In addition, 1 app offered screening to train the chatbot to learn which intervention strategies would be most relevant for each user.

#### Self-monitoring

Of the 36 apps, 22 (61%) offered functionality entailing tracking feelings (8/22, 36%), mood (11/22, 50%), emotions (1/22, 5%), or mood and anxiety levels (2/22, 9%). A diverse range of designs was used for self-monitoring. Common modalities included the use of emoticons or tags for different feelings, an avatar that changes based on interaction with it, and scales used to rate the intensity of different emotions. Colors were used to indicate the intensity of feelings and text to support the meaning of different emoticons. A total of 13 apps supported the entry of additional information related to the selected feeling, such as situations (4/13, 31%), factors (5/13, 38%), thoughts (3/13, 23%), or journal entries (1/13, 8%). The number of options for the selection of feelings differed among apps, with the majority presenting from 5 to 7 different feelings. Apps varied in how often users can track their mood and how mood tracking is presented. Tracking was unlimited and user-initiated in 20 apps. One app allowed 2 different types of tracking: daily tracking of worry level presented on the home page of the app, and mood tracking, which was triggered every time the user opened the chatbot feature of the app. All of the apps supported momentary tracking, with some allowing retrospective completion.

#### Visualization of Data Entries

A total of 30 apps provided ways to reflect on the data collected through the app. The apps offered a reflection on mood tracking data (20/30, 67%) or other kinds of data collected through the app (22/30, 73%), such as data from intervention tools. Color and emoticons were the most common elements used to display data on graphs. One app used cards showing a feeling and the factors related to that specific feeling. Other types of data included past entries in intervention tools (13/22, 59%), chatbot conversations (2/22, 9%), and frequency of app use (7/22, 32%). Customization options included the choice of the time range to display data (6/17, 35%), graph type (2/17, 12%), and selection of different variables (eg, mood vs sleep; 1/17, 6%). In addition, 3 apps provided weekly or monthly reports, including charts.

#### Gamification

Gamification techniques were integrated in 7 of the apps, including level upgrades (2/7, 29%), points (3/7, 43%), and badges (5/7, 71%) based on points earned from practicing different activities. One app mentioned that the reason for integrating points was to encourage regular use of the different activities. In addition, 1 app included a game to make negative thoughts concrete by knocking out negative feelings presented as cartoons.

#### Social Features and Support

All the apps were designed to function without professional guidance. Four of the apps offered the opportunity to involve health care experts by supporting access to counseling sessions either through the app (2/4, 50%) or over the internet with links to external websites in the premium version (2/4, 50%). In total, 15 apps allowed users to either share their data directly through the app with a therapist (2/15, 13%) or export and download data (13/15, 87%). Interestingly, 2 of the apps allowed for data sharing with wearable devices regarding mindfulness. Peer support was provided in five apps by integrating discussion and chat groups for various topics related to mental health. One of these apps required users to download another app for community features.

#### Immediate Support

Eight of the apps integrated a feature to provide additional momentary support. Seven of these apps provided a feature to access different intervention strategies to manage their mood and anxiety. A feature in one of the apps connected the user directly with an available professional through WhatsApp upon paying a “nominal” fee.

#### Crisis Support

In total, 14 apps offered in-app support via a feature that provided links to external support services and hotlines. One of the apps offered additional support by integrating a crisis feature that, apart from providing information on hotlines, created a safety plan and integrated a *grounding* technique for panic management. This feature presented different types of exercises (breathing, mindfulness, and physical exercises) through a chatbot. In another app, a chatbot presented resources on the screen when the user indicated a crisis by typing a specific word (ie, SOS). However, this feature was presented only when the user first used the app, potentially making it difficult to remember.

### Features Used for Delivery of CBT Treatment Elements

#### Delivery Format

Six apps used the format of one or more treatment programs, each comprising a number of modules. Four of these apps provided a single treatment program, whereas the rest provided access to multiple treatment programs at any time. Users had to complete each module in the program to access the next module. An alternative delivery format, used by 5 apps, was to use a chatbot to deliver intervention strategies; in 2 cases, the chatbot was used to deliver intervention strategies based on self-monitoring entries.

One app that integrated a chatbot provided intervention strategies only in the premium version of the app. Table 3 lists the CBT elements identified in the apps.

**Table 3:** Cognitive behavioral therapy evidence-based elements available in the apps.

App name	Psychoeducation	Self-monitoring	Cognitive	Behavioral	Relaxation skills
Bloom		✓^a^			
CBT Companion	✓	✓	✓•^b^	✓•	✓
CBT diary		✓			
CBT Journey			✓		
CBT MH		✓			✓
CBT diary		✓	✓		
CBT Tools	✓	✓	✓		
ClearFear	✓	✓			✓
CBT	✓		•	•	
CBT (2)	✓				
De-stressMe	✓	✓			✓
ezeCBT	✓		✓		
FearTools		✓	✓	✓	✓
FreeCBT			✓		
Happify					✓
Innerhour	✓	✓			✓
Life	✓	✓		✓	
Mindease		✓	✓		✓
Mindshift	✓	✓	✓	✓	✓
Moodfit	✓	✓	✓		✓
Moodnotes		✓•			
Moodpath	✓	✓			✓•
MoodSpace			✓		✓
Panic Pit Stop	✓				
Pocketcoach	✓	✓	•		✓•
Reflectly		✓			
Sanvello	✓	✓	✓•		✓
Stress & Anxiety Companion	✓		✓		✓•
Thoughts	✓		✓		
UpLift	✓	✓			✓
What’s up	✓	✓			
Woebot	✓	✓	✓	✓	✓
WorryKit			✓		✓
WorryTree				•	
Wysa		✓	✓		✓
Youper		✓	•		•

^a^Checkmark denotes an element offered in the free version of the app.

^b^Bullet symbol denotes additional elements offered in the premium version of the app.

#### Psychoeducation

Most apps delivered psychoeducation material (21/36, 58%) using a distinct feature integrated into the apps (16/21, 76%). Modalities for the delivery of psychoeducation included the use of text (11/21, 52%) separated into chunks (6/21, 29%) accompanied by illustrations (3/21, 14%). One of these apps additionally offered video and audio delivery for psychoeducation. Two apps delivered psychoeducation using a question-and-answer interface. Another 2 offered psychoeducation through a chatbot using multiple-choice topic selection or through users’ interaction with the app (eg, after check-in assessments, to explain different intervention strategies).

#### Cognitive Techniques

Cognitive techniques were supported by 56% (20/36) of apps and were mainly delivered through structured exercises, providing suggestions and prompts based on previously entered data. Many cognitive techniques, such as cognitive restructuring, were implemented through textual representations of thoughts, lists of thinking traps, and cognitive distortions. One app allowed audio entries as an alternative. Some apps supported a greater degree of interactivity; for example, 1 app supported identifying negative thoughts by dragging a finger over words, and a window would appear with a selection of thinking traps. Two of the apps used a chatbot to deliver a cognitive exercise aimed at reducing the burden on users by delivering content based on data collected during app use. More specifically, one of the apps remembers and presents a *rethink list* with unhelpful thoughts previously provided by the user. The user can remove or add thoughts from the list. The other app remembers and presents the most frequent distortions identified by the user in the past, allowing them to decide what to work on.

#### Behavioral Techniques

Behavioral techniques supported (7/36, 19%) included behavioral activation, exposure, and action planning. For example, 2 apps supported behavioral activation, focusing on positive rewarding activities. In one app (Woebot), users, after identifying negative thoughts, could schedule an activity and rate their feelings after activity completion. The other app used a chatbot, issued text instructions, and suggested activities to be completed based on the setting (home or outside).

#### Relaxation Skills

Most apps supported relaxation skills such as breathing (14/36, 39%), relaxation (10/36, 28%), and mindfulness exercises (17/36, 47%). Relaxation and mindfulness exercises were provided using audio tracks or text instructions and illustrations. In 22% (8/36) of apps, breathing exercises were delivered using a breathing indicator that visually represented a full breath. For example, a circle expands as users inhale and contracts as they exhale. Interestingly, one of the apps monitored heart-rate variability through the application of a finger to the phone camera to detect changes in detected *stress* during the breathing exercise.

### Tailoring: Customization and Personalization

#### Customization and Personalization Types

Table 4 shows the personalization offered across three types. We characterized customization as interface customization, treatment-oriented customization, and customization of data visualization.

**Table 4:** Tailoring of mobile apps.

App name	App-driven	Mood-driven	Notifications
Bloom	✓^a^		✓
CBT Companion			✓
CBT diary			✓
CBT Journey			✓
CBT MH			✓
CBT diary			✓
CBT Tools			✓
ClearFear	✓		
CBT			
CBT (2)			✓
De-stressMe			✓
ezeCBT			
FearTools			✓
FreeCBT			✓
Happify	✓		✓
Innerhour	✓	✓	✓
Life			
Mindease		✓	✓
Mindshift		✓	✓
Moodfit		✓	✓
Moodnotes			✓
Moodpath			✓
MoodSpace			
Panic Pit Stop			
Pocketcoach	✓	✓	✓
Reflectly			✓
Sanvello	✓	✓	✓
Stress & Anxiety Companion			✓
Thoughts			
UpLift	✓		✓
What’s up			
Woebot		✓	✓
WorryKit			✓
WorryTree		✓	✓
Wysa	✓	✓	✓
Youper	✓		✓

^a^Personalization offered.

#### Interface Customization

In total, 24 apps allowed users to customize the app either at the beginning (14/24, 58%) or through a menu (24/24, 100%). Customization options covered user profiles (nicknames, avatars, and profile photo), user interface appearance (themes, display options, animations, and language), and technical features covering notifications and location tracking.

#### Treatment-Oriented Customization

Three apps allowed the customization of different treatment elements from the settings menu, such as adding, deactivating, or changing the position of emotion management (1/24, 4%), hiding navigation arrows on a diary entry (1/24, 4%), enabling voice dictation for a thought diary (1/24, 4%), adding a therapist’s number (1/24, 4%), and stopping sharing data with a professional (1/24, 4%).

#### App-Driven Personalization

A total of 16 apps provided onboarding screens to educate users about the functions and benefits of each app. As part of this onboarding, nine apps allowed users to select the challenges or goals they wanted to work on (8/9, 89%) or request information about things that calm the user and phone numbers to be presented for emergency support (1/9, 11%). App-driven tailoring of app content was compulsory in 4 apps with no option to skip that step. Two apps did not provide information on the purpose of therapeutic tailoring.

#### Mood-Driven Personalization

Eleven apps suggested intervention strategies based on users’ self-monitoring data. The suggested intervention strategies were either randomized (3/11, 27%) or the same set of strategies (8/11, 73%) were presented to the users each time they tracked their mood, although in 1 chatbot-based intervention, the last activity practiced would also be suggested. Four of these apps presented intervention strategies only when low mood levels were indicated. One app allowed access to intervention strategies only in the premium version.

### Customization of Notifications

Notifications are used to prompt users to interact with apps. Nineteen apps integrated notifications and prompted access to the intervention content. Three apps offered notifications for tracking only in the premium version. Notifications were provided at fixed times by the user (8/19, 42%), fixed times by the app (4/19, 21%), randomly (3/19, 16%), or in combination (4/19, 21%). Users could customize notification timing for different purposes, such as tracking (16/19, 84%), accessing intervention strategies (7/19, 37%), or accessing other app features (7/19, 37%). Two apps allowed the customization of the reminder message.

## Discussion

### General Findings

Despite increased scholarly interest in the review of apps for a variety of mental health conditions [23-33], this is the first study to examine the delivery of content in CBT apps for anxiety, with a focus on engagement and tailoring features.

The reviewed apps targeted a variety of anxiety conditions and were not designed to tackle a specific anxiety disorder (eg, general anxiety disorder), which may limit the degree to which treatment could be tailored to individual users. There is also confusion around the appropriate age for these apps. Most apps were in the health and fitness category. However, some apps were advertised in the medical category without providing a medical disclaimer. These findings align with the results of a recent review that explored the functionality of top-rated apps for depression [24].

Three of the apps received awards from the marketplace (Google Play Store) based on the quality of their design and overall functionality. However, there are no clear guidelines on how an app’s content quality is assessed.

### Accessibility and Access

The results showed that accessibility was rarely considered when designing apps. The lack of consideration of accessibility during the design process potentially excludes a variety of users. To address accessibility issues, researchers have begun developing guidelines to gauge mobile app accessibility [48]. In addition, many apps cannot function or offer reduced functionality when an internet connection is not available. Several engagement features or full access to features are offered only in the premium version. Hence, valuable functionality is offered as a premium feature, and issues of access and cost should be addressed at the public health level.

### Engagement With Apps and Therapeutic Content

Overall, our results indicate that self-monitoring and visualization were relatively well explored by the apps in the sample in comparison with other feature types. Screening was provided using multiple-choice questions without involving any engagement feature for that purpose, which suggests an assumption by designers that screening itself could potentially be engaging. Several types of self-monitoring functionality are used to make the process more appealing and easier to use by minimizing the completion time. A minority of apps made use of basic gamification techniques such as points, badges, and level upgrades, in line with those found in a recent review of gamification in mental health [49].

### Human Support

Human support is a powerful mechanism for increasing engagement and enabling the tailoring of treatment. The most common engagement feature related to human support was the ability to share data through the app. A minority of apps provided some form of peer support feature, and for understandable reasons, the ability to contact a therapist was not offered in the free version of the apps. The therapeutic alliance is an important part of CBT treatment, and developing a relationship between a health care professional and a user could enhance the regular use of apps [50-52]. Further research is required to reveal the amount and type of clinician support needed to optimize the effectiveness of CBT for specific groups [8].

### Delivery of CBT Elements

CBT elements use various features, such as audio and video, illustrations, and interactive screens, to increase engagement. By using multimedia elements and interactive exercises, we can enrich the therapy experience and support learning [39]. In particular, behavioral techniques can be enhanced through sensing.

The CBT apps identified in this review were designed to support low-intensity CBT exercises. Although a structured treatment program was offered by a minority of apps, studies have shown that mobile apps do not provide a full course of CBT but are limited to specific techniques [53]. Limited support for clinician integration reduces the opportunity to use such apps in higher-intensity CBT.

### Customization and Tailoring

Although the classifications in the existing literature on tailoring health interventions focus on the delivery of tailored messages [54,55], this study explored tailoring more broadly in the context of anxiety apps that adjust content to suit different groups and user needs. The majority of apps allowed the customization of the interface. However, customization options for CBT elements were lacking, with only 3 apps supporting this. The results showed that app-driven therapeutic tailoring is restricted to push notifications and goal selection, although CBT involves the individualization of treatment.

Many apps offered tailoring based on self-monitoring data by delivering intervention strategies to users. The results indicated that either the same set of strategies is presented after reporting a particular score in self-monitoring or intervention strategies are presented at random. Thus, there are opportunities for more interactive and integrated approaches that incorporate both user preferences and mood-based recommendations**.**

### Sensors and Just-in-Time Intervention

Only one app used physiological sensing. Two of the apps provided helplines and resource information based on location data. This contrasts with the academic literature, in which much recent research concerns the vision of *just-in-time adaptive interventions* that allow the provision of intervention content when users are in need of support [12,56] and the interest in exploiting machine learning to optimize treatment delivery within mental health [57].

### Improving Delivery Within CBT Apps for Anxiety

Engagement with apps may be increased if we target mobile apps to specific types of anxiety disorders and user needs to enhance the degree of tailored content to individual user needs. Greater interactivity, for example, through increased user agency or better use of gamification, could enhance engagement [35]. Human support offered through the apps should be enhanced to increase engagement with low-intensity CBT apps and explore mechanisms for use in higher-intensity treatment. Designers should consider accessibility issues and offline functionality to increase the potential reach of these systems to wider populations.

### Evidence-Based Content and Effectiveness of Mobile Apps

The results showed that all apps claim in their description page to be designed based on validated psychological treatments. Previous research has shown that most apps are inconsistent with evidence-based treatments [30-32]. In addition, an app’s consistency with evidence-based treatment elements does not guarantee the efficacy and effectiveness of treatment elements delivered in an app-based format. Randomized controlled trials are considered to provide the most reliable evidence for the effectiveness of an intervention. However, these studies test the effectiveness of an intervention as a whole and do not examine the mechanisms that lead to improvement in one's mental health state. Further research is thus required to determine the most effective functionality for delivering evidence-based treatment content. We hope that the breakdown of app functionality in this study will be useful as a starting point for such efforts. In addition to effectiveness, future work could also examine proximal outcomes, such as the impact of different features and evidence-based components on engagement.

### Limitations

The search was based on US app stores and might not be representative of all anxiety apps available in the global market. This study focuses on apps integrating CBT elements and thus may have excluded potentially useful apps for people with anxiety. This review focused on investigating engagement features and tailoring of content; therefore, we did not evaluate the quality of content in the apps. We believe that by exploring the functionality of the apps for tailored and engaging delivery, our work complements existing research and can help inform future design efforts. Our results reflect consumer mobile apps available in the marketplace rather than the current state-of-the-art in research.

### Conclusions

Although apps integrate a range of functionalities and engagement features, the provision of these features is highly uneven. Self-monitoring and visualization are relatively well explored, with social features and human support rarely integrated. Our results show that, within currently available apps, accessibility is neglected, and tailoring options are limited. Furthermore, consumer apps do not currently take advantage of the technological capabilities of smartphones to deliver *just-in-time* interventions at opportune moments [12]. Future research should explore strategies for tailoring therapeutic content and involving clinicians to facilitate user engagement.

## Appendix

Multimedia Appendix 1 Spreadsheet of main coding elements.

## Abbreviations

CBT: cognitive behavioral therapy
